# Beyond Biosecurity by Taxonomic Lists: Lessons, Challenges, and Opportunities

**DOI:** 10.1089/hs.2022.0109

**Published:** 2023-12-18

**Authors:** Piers Millett, Tessa Alexanian, Kathryn R. Brink, Sarah R. Carter, James Diggans, Megan J. Palmer, Ryan Ritterson, Jonas B. Sandbrink, Nicole E. Wheeler

**Affiliations:** Piers Millett, PhD, is Executive Director, International Biosecurity and Biosafety Initiative for Science, Washington, DC.; Tessa Alexanian is Safety and Security Program Officer, iGEM Foundation, Paris, France.; Kathryn R. Brink, PhD, is a Postdoctoral Fellow, Center for International Security and Cooperation, at Stanford University, Stanford, CA.; Sarah R. Carter, PhD, is Principal, Science Policy Consulting LLC, Arlington, VA.; James Diggans, PhD, is Head of Biosecurity, Twist Bioscience, San Francisco, CA.; Megan J. Palmer, PhD, is Executive Director of Bio Policy & Leadership Initiatives and an Adjunct Professor, Department of Bioengineering; at Stanford University, Stanford, CA.; Ryan Ritterson, PhD, is Executive Vice President of Research, Gryphon Scientific LLC, Takoma Park, MD.; Jonas B. Sandbrink is a Doctoral Researcher, Nuffield Department of Medicine, University of Oxford, Oxford, United Kingdom.; Nicole E. Wheeler, PhD, is a Turing Fellow, Institute of Microbiology and Infection, University of Birmingham, Birmingham, United Kingdom.

**Keywords:** Taxonomy, Taxonomic list, Synthetic biology, Biological engineering

## Introduction

Taxonomic lists—that is, lists that give a set of species (eg, *Yersinia pestis*) or broader taxonomic groups (eg, SARS-related coronavirus) to be regulated—are a common tool used within biosecurity regimes for controlling access to pathogenic organisms and toxins. However, these lists are both too specific and too ambiguous for many of the uses to which they are applied. Their use is based on an assumption that taxonomy can be directly linked to biological function; however, other approaches, such as sequence lists or tools that predict function, may be more suitable to capture biological functions that cause concern. We are not the first to notice the problems with taxonomic lists; their shortcomings have been highlighted for over a decade.^1-4^

We now have a wealth of experience in using taxonomic lists. Advances in biological engineering have both further strained taxonomy-based control systems and offered new alternatives to them. For example, in their 2010 report,^3^ the US National Academy of Sciences noted that a system for predicting risk—a combination of the probability of harm occurring and the severity (consequences) of that harm, if it were to occur^5^—from genetic data was not possible then or in the “usefully near future.” We believe things have changed. Two US Intelligence Advanced Research Projects Activity programs—Functional Genomic and Computational Assessment of Threats (FunGCAT)^6^ and Finding Engineering-Linked Indicators^7^—have provided a drive toward functional prediction based on sequence, which is being applied directly to commercial DNA synthesis screening.^8^ Recent advances in protein structure prediction, combined with advances in generative models and high-throughput screening, promise to usher in a new era of protein design and formally remove the previously reliable assumption of a link between taxonomic origin and biological function.^9-11^

Recent policies and guidance have attempted to encode broad principles in place of taxonomic lists. At the international level, the latest World Health Organization *Laboratory Biosafety Manual*^12^ focuses on broad risk assessment* over taxonomic classification.^13^ However, at the national or institutional level, lists of species remain a common starting point for risk management.^†^^14-16^ The US *Recommended Policy Guidance for Departmental Development of Review Mechanisms for Potential Pandemic Pathogen Care and Oversight*^17^ covers any research that is “reasonably anticipated to create, transfer or use potential pandemic pathogens resulting from the enhancement of a pathogen's transmissibility and/or virulence in humans.”^18^ Several challenges with these policy experiments have already emerged, such as ambiguous scope,^19^ disagreements on what their text means in practice (eg, the term “endow or enhance pathogenicity” in export control rules^20^), a need for greater transparency in decisionmaking,^21^ and guidance on responsible communication.^22^

We do not advocate abandoning taxonomic lists entirely. Taxonomic lists are an excellent tool for controlling access to whole organisms, which is necessary for export controls^20,23^ and when possession of certain agents has been criminalized.^24,25^ They can also have implications for the practical implementation of other policies where the best control point is a whole organism. However, modern biotechnology increasingly makes use of parts of organisms to confer new traits to species, sometimes making the origin species of a single part a poor (or even misleading) proxy for risk assessment. New, complementary tools are needed.

We believe that taxonomic lists become less useful when used beyond their intended context. Although they were originally created to control physical access to organisms, taxonomic lists have been integrated into broader biosecurity policies and risk management processes. For example, gene synthesis screening routinely uses taxonomic classification as a core element of risk assessment. Commercial providers of DNA synthesis have noted the challenges of implementing taxonomy-based regulations for sequence-based products.^26,27^ Ascertaining whether a given order matches something to be controlled is difficult even for the most qualified companies. Taxonomic lists have also been used as a tool to narrow oversight of relevant biological research. For instance, although US dual use research of concern policies are intended to consider more indirect scenarios of misuse, including generation of “knowledge, information, products, or technologies,”^28^ the scope of the oversight is restricted to specific types of experiments, with a specific set of pathogens on a taxonomic list. This scope may not be a good fit for the work that warrants oversight; more than two-thirds of life scientists who declared that their work featured “dual-use potential” in a 2009 survey would not fall into the policy's scope.^29^

In this commentary, we highlight some of the challenges encountered with taxonomic lists in practice. We explore opportunities to make them more effective. We also stress the importance, and attempt an initial discussion, of how we might move beyond taxonomic lists to meet an evolving landscape of safety and security needs.

## A More Structured Approach to Designing Biosecurity Regimes

Too often, we use taxonomic lists because policymakers and regulators are familiar with them, but they need not be the default option. Not all policy purposes will require taxonomic lists, and we argue for a more deliberative approach (Figure).

**Figure.**
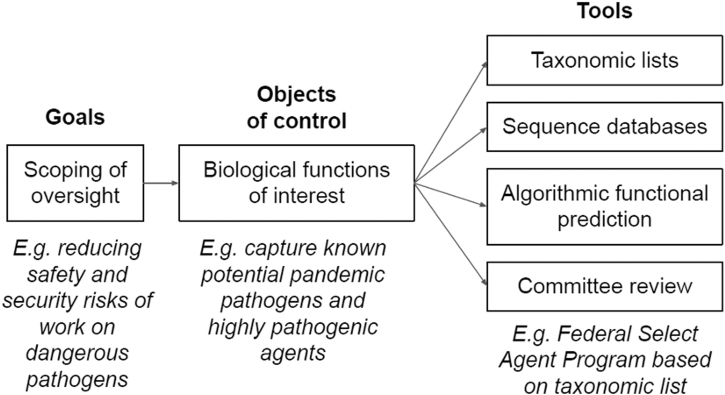
Tools for effective biosecurity policies. The goal and scope of a given regulation are a political decision. Once a scope has been set, regulators need to define the target objects with what functions should be targeted. What research objects feature these functions and are thus regulated can be defined using different tools.

The first step in developing a biosecurity regime should be a thorough understanding of its goals. What does the regime intend to accomplish? Is the goal to control *access* to particular materials, such as those likely to harm human health? Alternatively, the goal might be to control certain criminal *uses* of materials. Presumably, in all cases, the intent is also to avoid unintentionally impeding or unnecessarily restricting open, reproducible, and beneficial science and engineering. A regime that can articulate the ultimate goals and their underlying logic and principles is more likely to be able to be updated as assumptions are checked and revisited.

A second step is to translate the goals of the regime into biological functions to be controlled. A simple example is the goal of controlling pathogens that cause certain diseases, where the function to control is causing the disease. A more complex goal is controlling pathogens that meet thresholds for environmental stability, transmissibility, or virulence or for which vaccines and therapeutics do not exist. This goal recognizes the possibility of novel pathogens emerging that have similar biological properties to those known today. An even broader goal is to manage potential harms other than disease, such as those from gene drives (where the function would be to alter the inheritance of a certain trait) or antimateriel agents (where the function might be, for example, breaking down electronics). We assume that any regime's goal will focus on a subset of biological functions that pose a high risk of harm. This exempts other biological functions from control, thereby minimizing any undue impact on biological research and development.

The final phase is to identify which tools are best suited to the functions to be controlled. For example, a taxonomic list would be suited to a regime intended to control access to certain pathogens and to make their unauthorized possession a crime. By contrast, a regime aiming to control access to certain biological functions may need different tools, such as sequence databases. Recent efforts, such as FunGCAT,^6^ Secure DNA,^30^ SeqScreen,^31^ and the Common Mechanism for DNA Synthesis Screening,^32^ demonstrate the feasibility of compiling such databases. Emerging algorithms to predict function from sequence may be adopted when it becomes necessary to control access to genetic elements from novel or engineered sequences. We have identified a series of general rules when using any type of lists in these tools (Box).

Biosecurity regimes developed for different purposes in different locations by different institutions or by different communities will need different types of tools. Some tools may already exist (eg, taxonomic lists). Other tools (eg, sequence databases) may have been created for other purposes and may need to be reconfigured or curated for use in biosecurity regimes.^27^ Some tools needed by biosecurity regimes are not yet fully developed (eg, algorithms to identify harmful functions from primary DNA or protein sequences). When developing tools, it is vital to take into account the need to avoid unduly impeding biological research and development and to manage any information hazards generated.

## Improving Taxonomic Lists

There are at least 5 ways that taxonomic lists might be improved to increase and potentially extend their utility. Some of these would help address how we capture pathogenicity and virulence. Others will help address harms beyond traditional definitions of disease to address environmental disruption of antimaterial applications.

### Conduct More Regular and Structured Updates

Taxonomic lists must be regularly updated to respond to the emergence of novel pathogens, scientific discoveries, advances in engineering capabilities, and geopolitical factors that may impact threat models for misuse. Yet, in practice, policy updates can be slow to materialize. For example, a version of *Bacillus cereus* that features 2 plasmids similar to those determining the pathogenicity of *Bacillus anthracis* and causes anthrax-like disease was identified in 2010.^33,34^ Although most *B cereus* strains are not of concern, this strain, *B cereus serovar anthracis*, includes the concerning spore-forming and toxin-producing properties of *B anthracis*. Six years after this discovery, in 2016, the US Select Agents and Toxins List^25^ expanded to also capture “anthrax-like *B cereus.*”^35^ Many other taxonomy-based control lists have still not been updated to capture this pathogen, highlighting the importance of approaches and regimes that can be updated in a timely manner.^20,36^

### Include Functional Equivalence

Those responsible for maintaining taxonomic-based lists should commit to including functionally equivalent pathogens. This should be accompanied by annotating in their lists the criteria for the inclusion or exclusion of pathogens as well as the policy goals. This will make it easier to identify other pathogens that meet the same criteria based on functional equivalency. *B cereus serovar anthracis* is also a useful example of functional equivalency.

Including pathogens via functional equivalency could pose certain challenges. Including functional equivalence should not be used beyond control of whole organisms because it can be difficult, if not impossible, to link individual genetic elements as necessary or sufficiently causative of observed functional equivalency. In addition, including functional equivalence will require careful review for information hazards, for example, avoiding the publication of novel threat applications when explaining why a function is being included.

**Box. General Guidelines for the Use of Lists Within Biosecurity Regimes**

- **1.** **Determine whether a list of specific biological materials, a list of functions, or a set of broad principles is more appropriate for a given context.** Lists of biological organisms or sequences are useful tools for contexts in which narrow and specific reference to controlled agents is required, for instance for law enforcement. However, such lists only capture known concerns. Thus, in settings where novel risks are meant to be anticipated, lists of functions or sets of broader principles rather than lists of specific biological materials may be better suited to guide decisionmaking.
- **2.** **Clearly state whether a list is meant to be illustrative or exhaustive.** Better contexts around lists and their underlying principles need to be built. For a given list and underlying principles, it needs to be clear whether a list is solely indicative of the underlying principle or is exhaustive.
- **3.** **Regularly update lists based on new evidence.** A transparent process for regular, evidence-based updating of lists is required to address novel risks and changing circumstances. There is a need to develop processes to remove items from these lists; for example, when new vaccines or therapeutics become available or when a potentially pandemic pathogen becomes a pandemic pathogen.
- **4.** **Determine triggers for updating.** More work could be done to identify biological components and/or combinations of components that create risk. These, in turn, could be used to identify and update lists for novel threats, including traditional lists of pathogens. For example, a list of potential pandemic pathogens could be automatically updated if an agent was found to exhibit a given human receptor binding affinity and lack of preexisting population immunity.
- **5.** **Facilitate legitimate science.** Biosecurity tools that better match the potential to cause harm, and that are tailored for use with parts rather than whole organisms, tailored specifically for the purposes to which they are put, and updated continuously to keep pace with scientific understanding should reduce the restrictions or oversight burdens on scientists and researchers, compared with current approaches. Improving the fit between biosecurity tools and the concerns they are meant to address should facilitate legitimate science and not further restrict it.

### Adapt Taxonomic Lists to Consider Ease of Engineering

The misuse potential of pathogens depends not only on their inherent pathogenicity but also on whether they might be engineered to do additional harm. As a result, it may be desirable to use taxonomy-based lists to identify not just pathogens, but also which pathogens may be more readily misused because they are easier to work with or to engineer.^37^ For example, additional biosecurity oversight may be warranted for viruses with smaller genomes or those that are easier to “boot up” from naked genetic material to infectious agent. Our understanding of the science that underpins this is evolving rapidly. It will be vital that any oversight measures that consider the ease of engineering will need to be highly adaptive—they will need continual review and updating, as well as clear definitions and thresholds for what is meant by “ease” of engineering and how that ease is linked to increased risk of harm.

### Clarify Language Used in Taxonomic Lists

Taxonomic lists that define entries too broadly may unintentionally capture organisms that pose little potential for harm. For instance, a hypothetical list entry for “influenza A viruses” would group together both viruses with human pandemic potential and strains with mild transmissibility. This capturing of unintended strains may inadvertently hinder public health research. For instance, Australia Group export controls and national export controls outside the United States extend to “SARS-related coronaviruses,” potentially restricting researcher access to SARS-CoV-2 at a time when the virus was widely circulating in human populations and when countermeasure development was sorely needed. Clarifying the language used in taxonomic lists may assist implementers of biosecurity controls. For example, clarification as to whether SARS-CoV-2 is a “SARS-related coronavirus” for the purpose of export control has been made in some settings, such as by the US Department of Commerce, but is missing in other settings, such as the Australia Group common control lists.^38^

To avoid capturing large numbers of pathogens not relevant to biosecurity controls, some taxonomic lists have already had to develop additional criteria to focus controls beyond taxonomy. For example, attenuated strains used in many traditional vaccines have substantially different properties than their ancestors but still fall into the same taxonomic group. This has required clarification of rules. For example, the US Federal Select Agent Program features an avenue to apply for exclusions of attenuated strains of select agents, but these exclusions are granted on a case-by-case basis without clear guidelines that would enable automatic exclusion rather than review by the regulator.

Taxonomic lists that define entries too narrowly can fail to capture organisms that pose a harm equivalent to those listed. For example, the US Select Agent and Toxins list does not extend to all influenza A viruses but only captures 2 of its members (“1918 reconstructed influenza” and “highly pathogenic avian influenza viruses”).^‡^^39^ These rules have failed to capture other related viruses that may pose similar harms. For example, research to make H2N2 (a pathogen that has already caused a pandemic) more transmissible or virulent would not require enhanced US federal oversight.^40^

### Specify Controlled Taxons via Reference to Database Identifiers

Current lists often use species names (eg, “*Bacillus anthracis”* or “SARS-related coronavirus”), but databases attempting to capture the relationships between various taxonomic levels exist. They could serve as more specific reference points for these control lists. For example, entries in taxonomy databases, such as those maintained by the US National Center for Biotechnology Information, are hierarchical and very specific (eg, SARS-CoV-2 is taxonomy ID 2697049, which falls under taxonomy 694009 for SARS-related coronaviruses). If the US Department of Commerce really meant to control all SARS-related coronaviruses, expressing this as control of taxon 694009 provides greater clarity. Alternatively, if they only meant to control strains from the original SARS-CoV-1 and Middle East respiratory syndrome viruses, use of taxon IDs 2901879 and 1335626 would clearly indicate this intent. Furthermore, there have been efforts to develop taxonomic groups defined by reciprocal nucleotide identity, which streamlines and standardizes the definition of novel taxa and potentially increases their utility as part of a biosecurity control regime.^41,42^

## Moving From Taxonomic Lists to Sequence of Concern Databases

There are common constructs used in modern biotechnology that are not easy to classify using traditional taxonomy. Chimeric viruses, made up of fragments of different taxonomic origin, are frequently created for gene therapy, vaccines, and oncolytic viruses. Variations of such chimeric viruses could have pathogenic potential, yet multifamily chimeras may be excluded from the US Federal Select Agent Program even if they use select agent genes.^43^ Indeed, associated guidance states that “chimeras that are comprised of select agent and nonselect agent genes from the same virus family require careful review to determine select agent status,” admitting that chimeras defy taxonomic classification.^44^ Assessing the potential harm posed by a chimeric or recombinant virus can prove challenging: 1 case report details an HIV-1 infection acquired during routine experiments with supposedly noninfectious pseudoviruses.^45^ The report speculates that the worker was infected by an accidentally generated hybrid virion with expanded tropism and infectivity.

It may soon be possible to create novel organisms with potential for harm from “harmless” organisms without intentionally adding known genes of concern, whether through directed evolution^46^ or new multiplexed genome editing technology.^47^ Furthermore, some emerging technologies, such as *de novo* protein engineering, do not fit with an organism-based threat model and challenge taxonomic classification.

As it becomes increasingly possible to engineer organisms at the genetic level, sequence-based approaches may prove more effective than those based on whole organisms.

### Improving Sequence Databases Used to Implement Taxonomic Lists

Although the carefully curated taxon IDs managed by the US National Center for Biotechnology Information provide a reliable reference set upon which to make taxonomic classifications, the companion sequence databases, such as GenBank, are neither curated to the same degree nor were they designed to enable classification of a sequence's taxonomic origin. They were created to be and continue to serve as a tool for researchers to exchange data.^27^ As a result, these databases often include entries that have sequences that do not make taxonomic sense. For example, a researcher may upload a record consisting of a limited target sequence from a pathogen combined with the sequence of an unrelated reporter. The researcher may choose to label this record with the taxonomic identifier—for example, Ebola—perhaps because it is part of a set of similar sequences, each containing a snippet from a different pathogen. This means that other uses of this benign reporter gene may have a taxonomic best match to a database record classified as Ebola. Databases that provide reference genomes (ideally multiple references per organism) along with high-confidence individual gene records, explicitly for the purposes of sequence screening, would improve the accuracy and efficiency of taxonomic sequence screening while reducing the costs associated with addressing false positives.

### Sequences of Known Toxins and Virulence Factors

Given the desire to determine potential for harm based on functional characteristics rather than taxonomic categorization, databases should be developed to evaluate potential for harm based on functions inferred from DNA and protein sequences.^1,3^ Such a database would be immediately useful for screening synthetic DNA orders.^8,37^ A database of sequences of concern for this context would include sequences that encode listed toxins and genes known to “endow or enhance” pathogenicity from regulated pathogens. This would help clarify whether a specific sequence is subject to control or not. For example, at present, nearly 70% of virulence factors found in pathogenic bacteria—those that “endow or enhance pathogenicity”—are also found in nonpathogenic organisms.^48^ Such databases could be derived from existing, publicly available lists such as Victors^49^ and the Virulence Factor Database.^50^ The Nuclear Threat Initiative–World Economic Forum Technical Consortium for DNA Synthesis Screening is developing this type of database for its Common Mechanism.^32^

### Capturing a Wider Range of Sequences of Concern

There has been considerable interest in developing more powerful databases of sequences of concern that can capture a much wider range of functions, including those that are not found in known toxins and pathogens. The revised *Screening Framework Guidance for Providers and Users of Synthetic Oligonucleotides*,^51^ released in April 2022, recommends the use of such databases to flag potential harms from sequences of synthetic DNA, and several tools have been developed that could be used for this purpose.^31,52,53^ There is also interest in further expanding these tools to include machine learning-enabled predictions that can identify potential harms in unknown sequences. Users could also choose to include DNA sequences related to harms beyond pathogenicity or toxicity, such as those that could enable antimateriel applications.

### Limitations of Sequence of Concern Databases

Sequence of concern databases present their own challenges, especially related to curation and access. Sequence databases require expert curation both in their initial development and to keep pace with advances in pathogen research, which can be costly and require subjective judgments. Curation also requires clear annotations and communication about what sequences or functions should be considered of concern, by whom, and for what purpose (ie, regulatory control or merely increased attention on the ordering customer). An example of how this could be achieved is the Comprehensive Antibiotic Resistance Database, which combines manual expert curation (aided by machine learning-assisted text mining) with standardized vocabulary and inclusion criteria, such as requiring experimental validation of stated function, submitting the sequence to a public database, and publication on the mechanism in question.^54^ Concerns about misuse of these tools and information hazards contained in these databases are especially relevant as they expand to capture broader harms than those currently encompassed by existing public-facing lists.

## Enumerating Concerns in a Sequence- or Taxonomy-Agnostic Way

Biological systems can have emergent properties that pose concerns and are not easily described at the level of individual sequences. Some concerns arise from combinations of sequences. For example, sporulation is associated with weaponization potential because spores can be aerosolized and are resilient to harsh environmental conditions. Yet sporulation is a multistep biological process that relies on specific combinations of genes, making it difficult to capture through sequence-based approaches alone. In addition, synthetic biology tools can be used to combine otherwise innocuous sequences into biological systems that may pose biosecurity concerns, such as gene drives. Oversight mechanisms that regulate research at the level of biological functions, whether present naturally or endowed experimentally, could help to address some scenarios where sequence- and taxonomy-based approaches fall short.^55^ If research is regulated at the level of biological function, then lists, other guidelines, or algorithmic functional prediction tools may help review committees put these regulations in action.

A practical example of function-based approaches has been used in recent years for the International Genetically Engineered Machine (iGEM) competition. The competition uses a so-called “White List” to help identify biological materials that may pose a greater hazard and whose use requires prior approval and enhanced levels of oversight. In previous iterations, this approach addressed the use of specific organisms or biological parts in the competition. Since 2019, a series of activities has been included in the list requiring prior approval. Activities requiring additional hazard identification and risk management include all 7 experiments of concern, anything that notably alters risks associated with antimicrobial resistance, and anything that is likely to bias the inheritance frequency of a genetic marker in an organism's progeny, such as through the creation of a gene drive.^56^ In all these cases, it is the likely outcome that is subject to a rule and not a specific organism, part, or sequence. These functional definitions have been reviewed and revised considering practical experience in their implementation.^57^

Machine learning has enabled a dramatic shift in our understanding of proteins. Recently, Meta's ESMFold model has allowed the prediction of protein structure for over 600 million protein sequences.^11^ This, in turn, has enabled much greater fidelity in being able to design novel proteins for specific biological functions. Both being able to predict function from structure and being able to design for a specific function were once considered to be grand challenges. Progress in addressing these challenges is happening more quickly than anticipated. The same tools and approaches are now being applied to more complex biological challenges, such as use of “hallucination” to broaden potential protein design space, design of very small beta-barrel domains, or functional prediction of thermostability.^58-60^ It is possible that advances in characterizing biological systems will open the door to predicting and designing biological functions more broadly, thereby enabling biosecurity tools that look beyond just taxonomy or sequence. For example, generative protein modeling opens the door to much-needed screening tools based on protein structure or predicted binding targets.

Considerations beyond biological function should also be incorporated into biosecurity regimes where relevant. The specific public health, environmental, and economic impacts of a biological agent may be regionally and temporally specific. It may be undesirable or impossible to regulate access to a pathogen in areas where it is endemic or during an active global health crisis, and agricultural pathogens affecting certain crops may be less concerning in areas where that crop is not grown.

Any taxonomy- or sequence-agnostic approach should be carefully implemented to avoid introducing unmanageable levels of ambiguity. For example, whether research falls under the US potential pandemic pathogen care and oversight^18^ federal guidance is based on function, determined by whether it can be “reasonably anticipated to involve creation, transfer, or use of enhanced [potentially pandemic pathogens].”^61^ In this context, “reasonably anticipated” could mean either the researcher “wouldn't be surprised if” or alternatively “would be surprised if it did not.” The former captures much more research than the latter. Guidance on which approach was intended would help reduce subjectivity and assist in ensuring relevant research is being considered. Efforts by the National Science Advisory Board for Biosecurity to revise guidance for US rules and regulations provide an important opportunity for ensuring greater clarity.^62^

Equally, taxonomy- or sequence-agnostic approaches will need to be carefully developed and implemented to address their own potential for misuse. In some cases, the tools developed to manage potential for harm could assist those set on causing harm. For example, a machine learning tool that predicts potential harm from a sequence might be repurposed to identify novel harms or paths that circumvent existing biosecurity controls. We must ensure that biosecurity tools keep pace with these capabilities as they become available.

## Conclusion

We need to be more deliberative in the development and implementation of biosecurity regimes. Taxonomic lists of pathogens have provided a historical bulwark, but today, we have many more tools at our disposal. We must first understand the goal of the biosecurity regime and the biological functions that it is attempting to control; only then can we choose an appropriate tool to control these functions.

Biosecurity tools that better match the potential to cause harm, are tailored for use with parts rather than whole organisms and are updated continuously to keep pace with scientific understanding should reduce the oversight burdens on scientists compared with current approaches.

Taxonomic lists will remain important for certain purposes, such as controlling access to or criminalizing unauthorized possession of specific organisms. We have identified several ways in which taxonomic lists and their use inside biosecurity regimes can be improved.

We have also identified cases in which biosecurity regimes might make use of other types of lists. For example, efforts to control access to genetic elements or to manage risks associated with synthetic biology might benefit from databases of sequences of concern, both for implementing existing controls of known toxins and pathogens and for capturing broader functions of concern.

Ultimately, it may be desirable to move away from lists of organisms or sequences to more broadly describe the functions that are being controlled. There have already been efforts to integrate specific functions into biosecurity regimes. New tools are emerging to enable increasingly sophisticated implementation of function-based rules. Testing the efficacy of these tools, fostering agreement as to their utility, integrating them into regulations and oversight systems, and implementing their use will be challenging. We will need much more adaptive forms of risk management. There are notable regulatory and policy hurdles, as well as technical challenges, to be overcome.

In all cases, there is more research to be done. Taxonomic lists cannot be our only tool, especially as our understanding of biology and what disrupts its normal functioning grows. We can, and must, build better tools that are tailored to the realities of modern biotechnology.

## Notes

^*^
Risk assessment: A systematic process—quantitative or qualitative—of gathering information and evaluating the nature, probability, and magnitude of potential harms and determining the appropriate control measures to minimize or otherwise mitigate the risks.^5^

^†^
Risk management: The quantitative or qualitative forecasting and evaluation of the probability of harm occurring and subsequent consequences (risk assessment) together with the identification and implementation of technologies, measures, or practices to avoid or minimize their likelihood or impact.^5^

^‡^
Avian influenza strains are included for their danger to livestock, in particular chickens.
